# Author Correction: Evaluating analgesic efficacy and administration route following craniotomy in mice using the grimace scale

**DOI:** 10.1038/s41598-019-50826-5

**Published:** 2019-10-25

**Authors:** Chulmin Cho, Vassilia Michailidis, Irene Lecker, Chereen Collymore, David Hanwell, Mary Loka, Matthew Danesh, Christine Pham, Paige Urban, Robert P. Bonin, Loren J. Martin

**Affiliations:** 1 Dept. of Psychology, University of Toronto Mississauga, Mississauga, ON M9A1C5 Canada; 2 Cell and Systems Biology, University of Toronto Mississauga, Mississauga, ON M9A1C5 Canada; 3 Leslie Dan Faculty of Pharmacy, University of Toronto, Toronto, ON M5S 3M2 Canada; 4 Division of Comparative Medicine, Faculty of Medicine, University of Toronto, Toronto, ON M5S 1A8 Canada; 5 Research Oversight and Compliance Office, University of Toronto, Toronto, Canada

Correction to: *Scientific Reports* 10.1038/s41598-018-36897-w, published online 23 January 2019

The original version of this Article contained a typographical error in the spelling of the author Vassilia Michailidis, which was incorrectly given as Vassilia Michalidis. This has now been corrected in the PDF and HTML versions of the Article.

